# Diabetes

**Published:** 1892-09-17

**Authors:** 


### DIABETES.

We recently discussed some of the points in the treatment of diabetes which must be raised when we are confronted with the many varieties in the disease which clinical observation enforces us to recognise. We had rather drifted into the way of regarding diabetes mellitus as a clinical entity till the custom of examining the urine of our patients became more or less a universal routine practice, and, as a consequence, the occurrence of relatively frequent forms of glycosuria, which could not possibly be classed with true diabetes, became widely admitted. The physiological and clinical investigations of Claude Bernard and Pavy and others seemed to have formed a solid basis on which we could accurately formulate the pathogeny of diabetes, but of late we have been induced to recur to the older theory that diabetes is, sometimes at any rate, associated with, and dependent on, a pathological condition of the pancreas. It would appear that we must regard the true diabetes as only a convenient clinical term embracing several varieties of disease, which, while having essential features in common, are widely different in their pathogeny. In fact, the name diabetes will probably be used in reference to glycosuria in the same way as we now employ the term Bright's disease. In a most valuable and interesting communication to the Royal Medical and Chirurgical Society, Dr. H. J. Tylden* reviewed the more recently advanced views associating diabetes mellitus with pancreatic lesions, with an original observation on the pathology of the disease. Three lines of recent research promised to throw new light on diabetes: M. Lépine's glycolytic ferment in the blood; certain experiments which had been undertaken on the pancreas; and a renewed study of the morbid histology of the pancreas. The object of his paper was to describe some of the researches, briefly to criticise their methods, but, still more, to offer some criticisms upon the inferences which were being drawn from them. With regard to M. Lépine's work on the glycolytic ferment of the blood, Dr. Tylden asked that, granting the existence of this ferment in the blood drawn from animals, was it a vital phenomenon? The question could not be considered as settled, but there were grounds for supposing that it probably was. He asked if the diminution of this ferment in diabetes could be regarded as the cause of this disease. To this he advanced the following objections: (1) Perhaps all ferments were reduced in wasting diseases; (2) certain clinical facts warranted us in supposing that diabetes was due, not so much to failure of sugar destruction as to increase of sugar production in the system. As to his recent experiments on the pancreas, extirpation was found to be followed immediately by diabetes mellitus. Partial extirpation, injection of the pancreatic duct with irritants and tying of the pancreatic vessels; none of them produced permanent glycosuria, but led to cirrhosis of the pancreas, and were followed by azoturia, polyuria, and wasting. He asked if the glycosuria, which followed upon complete extirpation of the pancreas, was a result of the removal of the pancreas, or of some lesion incidental to the operation, and he thought that while there were difficulties in the way of either interpretation, probably the latter was the true answer to the question. Dealing with the morbid histology of the pancreas, he said that cirrhotic changes had been demonstrated in diabetic pancreases, and, no doubt, correctly; but before we accepted them as the cause of the disease, we must ask ourselves the following questions: 1. Was the lesion constant in diabetes? It certainly was not, some diabetic pancreases showing no departure in their histological characters, certainly none in the direction of cirrhosis. 2. Was it confined to diabetes, or had it wider relations with sundry other diseases? A microscopical examination of some fifty pancreases led to recognition of two forms of cirrhosis at least, the one coarse and interlobular, often seen in very wasted pancreases, the other finer and intercellular in pancreases not necessarily wasted. The latter form had been found to occur chiefly in patients who were the subjects of granular kidney. It was possible that the pancreatic change might throw light in some cases of chronic Bright's disease upon some of the symptoms of the disease, especially when we remembered that polyuria and wasting were two of the results of artificial cirrhosis of the pancreas. 3. Assuming the first two points, had we any reason to suppose that cirrhosis of the pancreas would, even if present, provoke glycosuria? As far as we knew we had not; experiments went to prove the contrary. As we could not answer one of the questions in the affirmative, we could not accept either the pancreatic hypothesis of diabetes, or M. Lepine's form of it.

* *Lancet*, January 30th, 1892.

